# Metagenome-Assembled Genome Sequence of Aphanizomenon flos-aquae Strain Clear-A1, Assembled from an Enrichment Culture

**DOI:** 10.1128/MRA.01124-20

**Published:** 2020-12-03

**Authors:** Tiffany Yemenian, Kyra M. Florea, J. Cameron Thrash, Eric A. Webb

**Affiliations:** a Department of Biological Sciences, University of Southern California, Los Angeles, California, USA; Georgia Institute of Technology

## Abstract

Aphanizomenon flos-aquae is a significant harmful algal bloom-forming cyanobacterial species. Here, we report the draft genome for a strain of *A. flos-aquae* (Clear-A1) from a harmful algal bloom enrichment culture. This metagenome-assembled genome (MAG) sequence comprises 4,452,466 bp in 60 contigs with a GC content of 37.1%.

## Abstract (precis)

Aphanizomenon flos-aquae is a significant harmful algal bloom forming cyanobacterial species. Here, we report the draft genome for a strain of *A. flos-aquae* (Clear-A1) from a harmful algal bloom enrichment culture. This metagenome-assembled genome (MAG) sequence comprises 4,452,466 bp in 60 contigs with a GC content of 37.1%.

## ANNOUNCEMENT

Enrichment cultures offer unique opportunities to understand microbial physiology and ecology because they provide laboratory representative genotypes and phenotypes for facilitating the study of natural ecological dynamics. We are broadly interested in characterizing the genetic potential of cyanobacterial harmful algal bloom (cyanoHAB) species. Here, we describe an *Aphanizomenon* metagenome-assembled genome (MAG) sequence obtained from Clear Lake, CA. This natural lake in central California is a recreation site, municipal freshwater source, and historical heritage site to native people and, despite this importance, commonly experiences toxic cyanoHABs containing *Aphanizomenon* (1, 2). The *Aphanizomenon* MAG sequence will aid in characterizing the evolutionary history and genomic potential of Clear Lake *Aphanizomenon*.

Colonies of *Aphanizomenon* were hand-picked from samples collected via surface tow at Clear Lake (lat 39.08946, long 122.83015) during a cyanoHAB in August 2019. The picked colonies were enriched in 50% BG-11_0_ medium and incubated at 25°C at 100 μmol Q/m^2^/s under a 12:12-h light/dark cycle for 7 months without added NaNO_3_ to enrich for diazotrophs. Additional medium was added every 2 weeks to sustain growth. A single enrichment culture was chosen for metagenomic sequencing. Biomass was filtered onto 25-mm-diameter 8-μm polycarbonate filters, and DNA was extracted using a DNeasy PowerBiofilm kit (Qiagen, Hilden, Germany). Before starting the kit protocol, biomass was washed into the bead-beating tubes provided and then subjected to 5 rounds of liquid N_2_ freeze-thaw cycles. The samples were then incubated with a proteinase K solution (25 μl of 25 mg/ml) overnight at 55°C. After incubation, the rest of the extraction was performed following the manufacturer’s protocol. The DNA quality was verified using Tris-borate-EDTA (TBE) gel electrophoresis and NanoDrop UV-visible (UV-Vis) spectroscopy. The DNA content was quantified using a Qubit spectrofluorometer (Thermo Fisher Scientific, Waltham, MA). After library preparation with a NEBNext DNA library prep kit according to the manufacturer’s recommendations, 1 Gbp of Illumina paired-end (PE) 2 × 150-bp sequencing was performed by Novogene (Nanjing, China) with 300-bp inserts, resulting in 11,285,566 reads. All quality control, assembly, and binning were performed on KBase using default parameters unless otherwise noted (3). The read quality was checked with FastQC v0.11.5 (4). The genome assembly was completed with metaSPAdes v3.13.0 (5); binning was performed using MaxBin2 v2.2.4 (6) and the genome annotated using PGAP (7). Taxonomy was assigned using GTDB-tk v1.1.1, run with “classify_wf” and the release 95 database (8). Functional annotation was estimated using FuncSanity as part of the MetaSanity wrapper (9).

The 4,452,466-bp MAG comprises 60 contigs (*N*_50_, 97,558 bp) with a GC content of 37.1%. Clear-A1 was estimated to be 99.89% complete with 0.22% contamination using CheckM v1.0.18 (with default settings) (10) and was identified as a strain of Aphanizomenon flos-aquae using GTDB-tk. Annotation predicted 4,015 coding genes. Metabolic reconstruction with the FuncSanity module of MetaSanity revealed predicted genes for oxygenic photoautotrophy via the Calvin-Benson cycle and nitrogen fixation.

### Data availability.

This whole-genome shotgun project has been deposited at DDBJ/ENA/GenBank under the accession number JACVZZ000000000. The version described in this paper is the first version, JACVZZ010000000. The BioProject accession number is PRJNA657201, and the reads are available in the SRA under accession number SRX8961728.
